# Alveolar Recess of the Maxillary Sinus Mimicking a Radicular Cyst: A Diagnostic Challenge and Systematic Literature Search

**DOI:** 10.1155/crid/2142677

**Published:** 2026-06-22

**Authors:** Alexandre Perez, Valerio Cimini, Cynthia Reymann, Nermine Joumaa, Tommaso Lombardi

**Affiliations:** ^1^ Unit of Oral Surgery and Implantology, Division of Oral and Maxillofacial Surgery, Department of Surgery, Faculty of Medicine, University Hospitals of Geneva, University of Geneva, Geneva, Switzerland, unige.ch; ^2^ Unit of Oral Medicine and Oral Maxillofacial Pathology, Division of Oral and Maxillofacial Surgery, Department of Surgery, Faculty of Medicine, University Hospitals of Geneva, University of Geneva, Geneva, Switzerland, unige.ch

## Abstract

**Background:**

Misdiagnosis on radiographic examination is a significant risk for dentists and medical practitioners. This review focuses on an anatomical variation of the maxillary sinus that can mimic a radicular (periapical) cyst. Knowledge of morphological variations, their prevalence, and the radiographic criteria distinguishing these variations from true lesions is essential to ensure accurate diagnosis and appropriate treatment and to prevent unnecessary or potentially harmful interventions.

**Objective:**

The aim of this study was to perform a systematic literature search of published cases and to present a clinical case illustrating a specific maxillary sinus variant, an enlarged sinus with a prominent alveolar recess that can mimic a periapical cyst on radiographs.

**Methods:**

After defining specific MeSH terms, a systematic literature search was conducted across three databases: Web of Science, PubMed, and Embase. Relevant articles reporting on enlarged maxillary sinuses with alveolar recesses resembling radicular cysts were identified and analyzed.

**Results:**

Four articles describing the presence of an enlarged maxillary sinus with an alveolar recess resembling a periapical cyst were extracted from the literature.

**Conclusion:**

Knowledge gaps regarding the alveolar recess of the maxillary sinus remain in the literature and should be addressed to avoid misdiagnosis. The findings also underscore the importance of three‐dimensional imaging, such as CT or CBCT, for accurate detection of this anatomical variant.

## 1. Introduction

Radiological examinations of the maxillofacial region are often performed incidentally when imaging the paranasal sinuses, eyes, neck, cervical spine, or brain. Given the wide anatomical variability in this region, understanding common anatomical variations is essential for accurate interpretation.

The maxillary sinuses are pyramidal‐shaped cavities located within the body of the maxilla. The posterior and the anterior walls form, respectively, the anterior boundary of the pterygopalatine fossa and the anterior aspect of the maxilla. The medial wall is defined by the inferolateral wall of the nasal cavity, and the superior wall forms the orbital floor. The inferior wall of the sinus lies adjacent to the alveolar process, which contains the roots of the maxillary dentition. Each sinus has four recesses: the infraorbital recess (superior), the zygomatic recess (lateral), the alveolar recess (inferior), and the palatine recess (a variable extension of the alveolar recess) [1, 2] (Figure 1). It is important to note that the shape and size of the maxillary sinuses vary significantly among individuals [3].

**Figure 1:**
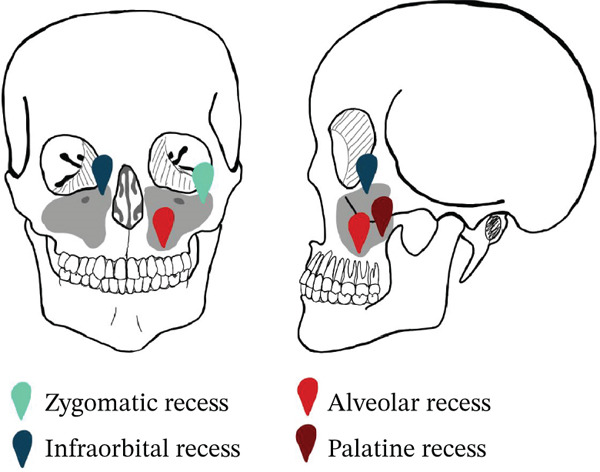
Location of the maxillary sinus recesses.

The alveolar recess is more frequently observed in edentulous patients and is intimately related to the roots of premolars and molars. Its prevalence has been reported to range from 18.8% [4] to as high as 50% of the population [5].

The present article reports an incidental finding of an alveolar recess during a routine dental radiographic examination. The objective is to describe this unusual case and review similar cases reported in the literature, thereby expanding current knowledge and providing guidance for clinicians and radiologists who may encounter such variations. Although this anatomical variation is relatively common, the case underscores the importance of careful radiographic assessment and, when necessary, consultation with imaging experts to avoid unnecessary or potentially harmful interventions.

## 2. Methods

### 2.1. The Identifying Question

This study, consisting of a case report accompanied by a systematic literature search, was aimed at addressing the following question:

What is the current state of knowledge regarding maxillary sinus enlargement in the form of an alveolar recess that may resemble a radicular (periapical) cyst, based on the available literature? We have defined the literature component as a systematic search of published cases to accurately reflect the nature of the available evidence, distinguishing it from a comprehensive systematic review.

### 2.2. Search Strategy

An electronic search was conducted (C.R. and V.C.) for all relevant articles published in PubMed, Embase, and Web of Science, with no restriction on publication date. The last search was performed on November 12, 2025. Data were extracted manually by the authors who conducted the electronic search. While this systematic search was conducted adhering to PRISMA‐ScR principles, it should not be classified as a formal scoping review, given the limited and case‐based nature of the identified literature [6].

The screening process involved assessing titles, abstracts, and full texts to determine eligibility for inclusion. The search strategy was structured using Medical Subject Headings (MeSH) terms, including “anatomic variation” and “maxillary sinus,” as well as Emtree thesaurus terms, including “anatomical variation” and “maxillary sinus.” Additional keywords were included in the search equation (Table 1).

**Table 1:** Search equations used for this systematic literature search.

PubMed	((“Anatomic Variation”[Mesh] OR “anatomic∗ varia∗”[tiab] OR “morpholog∗ varia∗”[tiab] OR “normal varia∗”[tiab]) AND (“Maxillary Sinus”[Mesh] OR “antrum of highmore”[tiab] OR “highmore antrum”[tiab] OR “maxilla sinus”[tiab] OR “maxilla sinuses”[tiab:~0] OR “maxillar sinus”[tiab] OR “maxillar sinuses”[tiab] OR “maxillary antrum”[tiab] OR “maxillary ostium∗”[tiab] OR “maxillary sinus”[tiab] OR “maxillary sinuses”[tiab] OR “sinus maxillaris”[tiab] OR “upper jaw sinus”[tiab:~0] OR “upper jaw sinuses”[tiab:~0]))
Embase	(“anatomical variation”/exp OR “anatomic∗ varia∗”:ab,ti,kw OR “morpholog∗ varia∗”:ab,ti,kw OR “normal varia∗”:ab,ti,kw) AND (“maxillary sinus”/exp OR “antrum of highmore”:ab,ti,kw OR “highmore antrum”:ab,ti,kw OR “maxilla sinus”:ab,ti,kw OR “maxillar sinus”:ab,ti,kw OR “maxillary antrum”:ab,ti,kw OR “maxillary ostium∗”:ab,ti,kw OR “maxillary sinus”:ab,ti,kw OR “sinus maxillaris”:ab,ti,kw OR “upper jaw sinus”:ab,ti,kw)
WOS	((“anatomic variation” OR “anatomic∗ varia∗” OR “morphologic∗ varia∗” OR “normal varia∗”) AND (“maxilla∗ sinus∗” OR “antrum of highmore” OR “highmore antrum” OR “maxillary antrum” OR “maxillary ostium∗” OR “sinus maxillaris” OR “upper jaw sinus∗”))

The Population, Concept, and Context (PCC) framework, as proposed by Pollock et al. [7], was used to guide article inclusion in this systematic literature search of published reports. The following parameters were defined:

• Population (P): living humans and cadavers.
• Concept (C): anatomical variations of the maxillary sinus (e.g., enlarged sinus or prominent alveolar recess) that may mimic a radicular (periapical) cyst.
• Context (C): maxillary sinus.

### 2.3. Inclusion Criteria

Studies reporting the discovery of an enlarged maxillary sinus with an alveolar recess mimicking a radicular cyst on two‐dimensional imaging were included. Both orthopantomograms (OPGs) and intraoral radiographs were considered. Eligible study designs included cohort studies, case reports, randomized controlled trials, and nonrandomized controlled trials. In addition, meta‐analyses and systematic reviews were included when available, using a forward snowballing method, which involves analyzing each article cited within a potentially relevant study.

### 2.4. Exclusion Criteria

Studies were excluded if they met any of the following criteria:

- Abstracts only
- Non–peer‐reviewed articles
- Conference papers
- Studies that did not investigate anatomical variations
- Studies that did not report radiographic findings simulating periapical pathology

## 3. Results

A total of 1591 articles were retrieved from the database search: 345 from PubMed, 474 from Embase, and 772 from Web of Science. Duplicates were removed using Zotero′s automatic duplicate detection tool and by manual review, resulting in 1.082 unique articles.

Following duplicate removal, titles and abstracts were screened to identify studies that met the PCC parameters. After this initial screening, 157 studies were selected for full‐text review. Ultimately, four studies met the inclusion criteria: three case reports and one review.

The flow of the database search and selection process is illustrated in Figure 2, and the results are summarized in Table 2.

**Figure 2:**
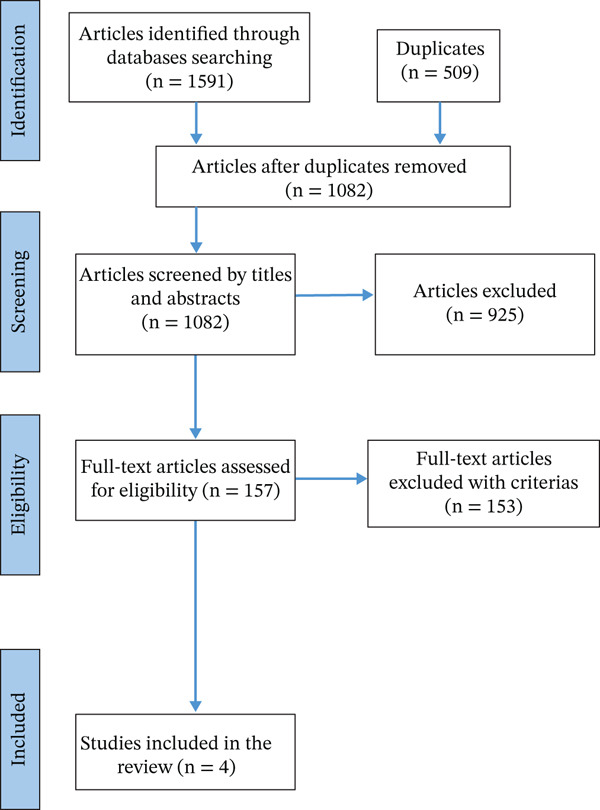
PRISMA flowchart.

**Table 2:** Summary of the included articles and their characteristics.

Authors and year	Type of study	Cases (*n*)	Gender	Age	Tooth concerned	Size of extent of the radiolucency (mm)	Treatment	Type of radiograph
Basnet et al., 2005 [8]	Case report	1	Female	19	Left upper canine to the first left molar	> 10	No treatment	Intraoral apical radiograph, panoramic radiograph, and ultrasound with color power Doppler examination
Sekerci et al., 2013 [9]	Case report	2	Male	25	First upper right molar	13.2 × 14.2 × 9.3	Endodontic retreatment	Panoramic radiograph and CBCT
			Female	29	Left upper second premolar and first molar	7.3 × 6.7 × 4.7	No treatment	Panoramic radiograph and CBCT
Poleti et al., 2014 [10]	Case report	1	Male	27	Left upper first and second premolars		No treatment	Panoramic radiograph and CBCT
Ito et al., 2022 [11]	Review	1	Male	30	First and second upper right molars			Panoramic radiograph and CBCT
Our case	Case report	1	Male	50	Upper left second premolar and first molar	5 × 5 × 5	No treatment	Panoramic radiograph and CBCT

## 4. Case Presentation

A man in his 50s was referred to the Oral Surgery and Implantology Unit for an annual check‐up by his transplant physician, with the primary objective of identifying potential sources of infection. The patient had a complex medical history, including a previous lung transplantation, kidney failure, Gilbert′s disease, and diabetes. He had been treated by his dentist for many years and had a long history of caries requiring composite restorations and fillings. One year prior to the present consultation, the patient had undergone a facial computed tomography (CT) scan in the context of maxillary sinusitis, which was treated by endonasal middle meatal antrostomy performed by his ENT specialist.

On the day of consultation, the patient reported no signs of infection or other symptoms.

Intraoral examination revealed carious lesions and several failed restorations. There was no history of jaw trauma or previous jaw surgery, and the patient was unaware of the presence of the radiolucent area discovered during the dental examination. No sensory or motor deficits were observed. Teeth 24, 25, 26, and 27 were asymptomatic, all responded positively to cold vitality testing, and periodontal probing was within normal limits.

The incidental discovery of a radiolucent area in the upper‐right quadrant required further investigation. The appearance of the image initially suggested a radicular cyst related to a maxillary molar. However, after reviewing the previously performed CT scan, the radiolucency was determined to correspond to an anatomical variation of the maxillary sinus, namely, an alveolar recess. The patient furnished written consent for publication of this case and accompanying images.

### 4.1. Investigation

Clinical examination confirmed the vitality of Teeth 24 through 27 and the absence of periodontal disease. The patient reported no pain in the upper‐right quadrant.

The first radiographic examination performed was a panoramic dental radiograph (Figure 3), which revealed a round, well‐defined radiolucent area measuring approximately 5 mm, located between Teeth 25 and 26. Given the patient′s long‐term dental care and rigorous follow‐up over the years to prevent infectious complications, radiographic examinations had been performed regularly.

**Figure 3:**
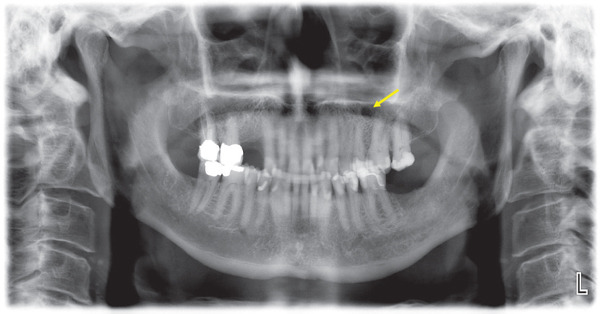
Panoramic radiograph (orthopantomogram) displaying a well‐defined radiolucency in the posterior maxilla. The yellow arrow identifies an extensive alveolar recess of the maxillary sinus mimicking a periapical lesion.

As part of the management of maxillary sinusitis by his ENT specialist, a CT scan had been performed 1 year earlier (Figure 4). Comparison of the panoramic radiograph with the head and neck CT images showed no lesion of the maxillary bone. Instead, the CT scan revealed a large air‐filled space along the inferior wall of the maxillary sinus.

**Figure 4:**
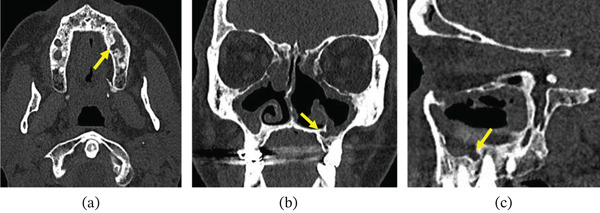
Cone‐beam computed tomography (CBCT) images in (a) axial, (b) coronal, and (c) sagittal planes. The yellow arrows highlight the alveolar recess. The images demonstrate anatomical continuity with the sinus cavity, thereby confirming its nonpathological nature and ruling out a cystic lesion.

The diverticular configuration of the sinus wall, combined with the anatomical morphology of the maxillary sinus, contributed to the cyst‐like appearance observed on the panoramic dental radiograph.

### 4.2. Differential Diagnosis

The initial panoramic dental radiograph evoked a cystic‐type lesion in the left posterior maxilla. The round shape and well‐defined margins were consistent with a radicular cyst. However, pulp vitality testing ruled out pulp necrosis and, consequently, an inflammatory cystic lesion.

Subsequent CT imaging revealed no cystic lesion. Instead, the CT scan demonstrated an anatomical variation of the maxillary sinus. By following the contours and morphology of the sinus, the inferior limit of the sinus cavity and its atypical extension could be clearly identified. This anatomical configuration corresponds to an alveolar recess of the maxillary sinus.

### 4.3. Treatment

No treatment was necessary for the patient, and no radiographic follow‐up was required.

### 4.4. Outcome and Follow‐Up

Comparison with previous panoramic dental radiographs obtained over the years showed no changes in the radiographic appearance of the affected area. The availability of earlier follow‐up radiographs and previously performed CT scans allowed assessment of the evolution of the radiolucent image over time.

A panoramic radiograph taken 8 years earlier demonstrated no change in the region. Consequently, no treatment was required, and no additional radiographic follow‐up was deemed necessary.

## 5. Discussion

The maxilla consists of a main body and four processes: frontal, zygomatic, palatine, and alveolar [1, 12]. The body of the maxilla contains the maxillary sinus, which presents four recesses: the infraorbital, zygomatic, alveolar, and palatine recesses. The shape and size of the maxillary sinus may vary considerably between individuals, and even within the same patient, anatomical differences may exist between the right and left sides [13].

Several anatomical variations of the maxillary sinus have been described, including those presented by Ito et al., among which the alveolar recess represents a specific variant characterized by an extension of the maxillary sinus toward the alveolar process. The alveolar recess is often bilateral but may mimic a cystic lesion on radiographs when present unilaterally [12].

According to Earwaker, this anatomical variation is more frequently observed in edentulous patients, in whom extensive pneumatization of the alveolar bone may occur [4]. However, the articles identified in the present literature search also reported dentate patients of various ages presenting with an alveolar recess. However, despite being an anatomical variation, only a limited number of reported cases have described its radiographic confusion with radicular cysts on OPG or intraoral imaging.

Indeed, the six cases identified in the literature exhibited radiographic features typically associated with cystic lesions, including a well‐defined cortical border and a radiolucent internal structure [14]. Most reported cases corresponded to an enlarged maxillary sinus with a prominent alveolar recess, whereas only isolated cases demonstrated additional alveolar bone pneumatization.

### 5.1. Clinical Implications and Diagnostic Pitfalls

From a diagnostic perspective, the main challenge lies in the differentiation between an alveolar recess of the maxillary sinus and true periapical radiolucent lesions. This distinction is critical to avoid unnecessary endodontic or surgical interventions.

Radicular cysts typically arise from pulpal necrosis and are associated with nonvital teeth, presenting as well‐defined periapical radiolucencies. Periapical granulomas represent chronic inflammatory lesions characterized by granulation tissue without an epithelial lining and are generally smaller in size [15, 16].

Conventional two‐dimensional imaging, including panoramic and intraoral radiographs, represents a projection of three‐dimensional anatomical structures onto a flat plane. As a consequence, anatomical superimposition occurs, particularly in the posterior maxilla, where the maxillary sinus is closely related to dental apices. This projection phenomenon may result in the alveolar recess of the maxillary sinus being superimposed over the root apices of maxillary teeth, creating a pseudocystic radiolucent appearance.

Due to the absence of depth information in two‐dimensional imaging, this anatomical air‐filled space may mimic a well‐defined periapical lesion, especially when corticated sinus walls project over dental structures. This radiographic limitation explains the frequent misinterpretation of the alveolar recess as a radicular cyst [17].

In contrast, the alveolar recess of the maxillary sinus is a normal anatomical variant corresponding to a physiological inferior extension of the sinus cavity. On two‐dimensional imaging, this structure may simulate a periapical radiolucency when projected over dental roots, despite maintaining direct continuity with the maxillary sinus on cross‐sectional imaging [15, 16].

### 5.2. Structured Radiological Comparison

Key radiographic and clinical features distinguishing these entities are summarized as follows:

- Tooth vitality: nonvital in radicular cysts versus vital in anatomical variation
- Nature: pathological lesion versus anatomical extension
- Imaging appearance: isolated periapical radiolucency versus continuity with maxillary sinus
- CBCT/CT: cystic lesion versus air‐filled sinus extension
- Management: endodontic/surgical treatment versus no treatment required

### 5.3. Clinical Consequences of Misdiagnosis

Misinterpretation of this anatomical variation frequently leads to unnecessary endodontic interventions. As documented in previously reported cases, initial misdiagnoses of periapical pathology often result in ineffective treatments before the correct radiological nature is identified. Consequently, a rigorous clinicoradiological correlation is essential to prevent such diagnostic errors.

### 5.4. What Is New in This Study

While the alveolar recess is a known anatomical variant, its role as a mimicker of periapical pathology remains poorly documented.

To the best of our knowledge, this study provides the first structured synthesis of published cases describing enlarged maxillary sinuses with prominent alveolar recesses simulating periapical cystic lesions. In addition, we propose a pragmatic diagnostic workflow integrating clinical examination, pulp vitality testing, and CBCT analysis to reduce diagnostic ambiguity and prevent unnecessary interventions.

### 5.5. Case‐Based Synthesis of the Literature

The first case was described by Basnet et al. in 2005. They reported a large, well‐defined radiolucent area on an intraoral radiograph at the apices of the left maxillary canine extending to the first molar, resembling a radicular cyst, in a 19‐year‐old woman. Clinical examination revealed carious lesions, but all involved teeth responded positively to vitality testing. An OPG demonstrated an enlarged maxillary sinus extending to the roots of the left maxillary teeth, and ultrasonographic examination revealed a hyperechoic area with posterior dirty shadowing, consistent with an air‐filled cavity [8]. This case is comparable to the present case in terms of preservation of pulp vitality and cyst‐like radiographic appearance. However, a key difference lies in the diagnostic workflow, as cross‐sectional imaging was not initially performed and ultrasound was required as an adjunctive modality, whereas in the present case, CT imaging was already available and allowed immediate anatomical confirmation.

The second report, published in 2013 by Sekerci et al., described two cases of anatomical variations mimicking periapical cysts.

The first case involved a 25‐year‐old man referred by a dentist for the evaluation of a suspected periapical lesion at the apex of the upper‐right first molar prior to prosthetic management. Clinical examination revealed several carious lesions in different regions. Panoramic radiography showed a well‐defined radiolucent area with radiopaque borders at the apex of the endodontically treated maxillary right first molar, measuring 13.2 × 14.2 × 9.3 mm. The initial diagnosis was an inflammatory periapical cyst, and endodontic retreatment was performed. After several months of follow‐up, no radiographic changes were observed, and the patient was referred to the Department of Oral and Maxillofacial Radiology. Pulp vitality testing showed that all teeth, except the treated molar, were vital. CBCT imaging revealed a maxillary sinus extending inferiorly to the roots of the maxillary first molar, consistent with an alveolar recess. Compared to the present case, this report highlights a more advanced diagnostic error, as an invasive endodontic retreatment was performed before CBCT confirmation, whereas in the present case, early access to cross‐sectional imaging prevented unnecessary treatment. This underscores the importance of early CBCT evaluation in equivocal cases.

The second case described a 29‐year‐old woman referred for periodontal management and routine dental examination. Panoramic radiography revealed a unilocular radiolucent lesion with a sclerotic border at the apices of the left maxillary second premolar and first molar, measuring 7.3 × 6.7 × 4.7 mm. All teeth in the affected area responded positively to vitality testing. CBCT was performed to determine the exact location of the lesion and demonstrated an extended alveolar recess of the maxillary sinus [4]. This case is highly consistent with the present case in terms of clinical presentation, preservation of pulp vitality, and final CBCT‐based diagnosis. However, in the present case, previous CT imaging allowed retrospective confirmation of lesion stability over time, further supporting its anatomical nature.

In the last case report of the present series, Poleti et al. described in 2014 the case of a 27‐year‐old man referred for evaluation of a panoramic radiograph showing a radiolucent area with radiopaque borders in the apical region of the left maxillary premolars. Pulp vitality testing was positive for all teeth. The initial differential diagnosis included an anatomical variation of the maxillary sinus or a bony lesion. CBCT imaging was performed to confirm the diagnosis and revealed a specific configuration: a prominent alveolar recess terminating in a septum [10]. Unlike alveolar recesses, maxillary sinus septa are well‐documented anatomical variations, with several systematic reviews reporting a prevalence of up to 41% [18, 19]. Septa are also recognized anatomical variants that may mimic periapical pathology [14]. Compared to the present case, this report demonstrates similar diagnostic uncertainty on two‐dimensional imaging; however, the presence of a sinus septum introduces additional anatomical complexity, potentially increasing the risk of misinterpretation. In both cases, CBCT was essential to establish the correct diagnosis, confirming the limitations of panoramic imaging.

In the article by Felix et al., emphasis was placed on the alveolar recess as a potential diagnostic pitfall. Although the reported case did not explicitly mimic a maxillary cyst, it highlighted the presence of this anatomical variation and its relevance in radiographic interpretation [20]. This report further supports the concept that the alveolar recess may be overlooked or misinterpreted on conventional imaging, reinforcing the need for careful radiological assessment.

Finally, in 2022, Ito et al. described normal variants of the orofacial region, including anatomical variations of the maxillary sinus. They reported that an enlarged maxillary sinus with a prominent alveolar recess may imitate a periapical cyst when unilateral. Two panoramic radiographs were presented to illustrate this variation: one involving a 63‐year‐old woman with an oval radiolucent lesion on the right side of the maxilla, confirmed to be a postoperative cyst, and another involving a 30‐year‐old man with a smaller, round radiolucent area with a well‐defined radiopaque border at the apices of the right maxillary first and second molars. In the latter case, CBCT imaging confirmed the presence of an alveolar recess associated with an enlarged maxillary sinus [12]. This report is closely aligned with the present case, particularly in demonstrating that an alveolar recess may present as a well‐defined radiolucency on panoramic imaging and can only be reliably distinguished from pathology using CBCT.

### 5.6. Diagnostic Criteria and Clinical Decision Framework

To facilitate clinical interpretation and improve diagnostic accuracy, a structured comparison of the key diagnostic features distinguishing a radicular cyst from a maxillary sinus alveolar recess is presented in Table 3.

**Table 3:** Key diagnostic criteria distinguishing radicular cysts from the maxillary sinus alveolar recess based on clinical, radiographic, and CBCT findings.

Diagnostic feature	Radicular (periapical) cyst	Maxillary sinus alveolar recess
Etiology	Inflammatory odontogenic lesion secondary to pulp necrosis	Normal anatomical variation (sinus extension)
Tooth vitality	Nonvital tooth (loss of pulp vitality)	Vital teeth in the adjacent region
Clinical symptoms	May present with pain, tenderness, or swelling (not always)	Asymptomatic
Radiographic appearance (2D imaging)	Well‐defined periapical radiolucency, often round or oval, associated with the root apex	Well‐defined radiolucency mimicking a periapical lesion, often superimposed on roots
Cortical border	Corticated border possible, related to lesion expansion	Thin sinus cortical outline continuous with the sinus wall
Relationship to root apex	Centered on apex with loss of lamina dura	Superimposed over the apex without a true apical origin
CBCT/CT findings	Cystic lesion with bone expansion and/or cortical thinning/disruption	Air‐filled cavity continuous with the maxillary sinus, without bone destruction
Continuity with the sinus cavity	Absent	Present (direct anatomical continuity)
Effect on the surrounding bone	Possible bone remodeling or expansion	No pathological bone alteration
Management	Endodontic treatment ± surgical enucleation	No treatment required
Prognosis	Requires intervention; may progress if untreated	Benign anatomical variant, stable over time

A rigorous clinicoradiological correlation is essential to avoid misdiagnosis of maxillary sinus anatomical variations as periapical pathology. In this context, CBCT plays a pivotal role in confirming sinus continuity and should be considered the imaging modality of choice in cases where two‐dimensional radiographs are inconclusive.

## 6. Conclusion

The literature search showed that although the alveolar recess is a rather frequent anatomical variation of the maxillary sinus, important gaps remain regarding its true prevalence and its impact on the clinical management of lesions with a cyst‐like appearance.

Current evidence confirms that the maxillary sinus may present several extensions, referred to as recesses, but one of the main challenges encountered in this review was the heterogeneity of studies addressing anatomical variations of the maxillary sinus.

These findings should be interpreted with caution, given the limited and heterogeneous nature of the available literature. Nevertheless, a heightened awareness of anatomical variations, such as the alveolar recess, is essential for clinicians to prevent diagnostic pitfalls and avoid unjustified or potentially iatrogenic interventions.

While panoramic and intraoral radiographs provide initial diagnostic clues, three‐dimensional imaging—specifically CBCT—is indispensable for establishing an accurate diagnosis in equivocal situations. Ultimately, this study underscores the necessity of a rigorous clinicoradiological correlation, with histological examination remaining the gold standard for definitive confirmation when indicated.

## Author Contributions

Conceptualization: A.P. and T.L. Methodology: A.P., T.L., and V.C. Software: A.P., N.J., and C.R. Validation: A.P. and T.L. Formal analysis: A.P. and V.C. Investigation: A.P., V.C., and C.R. Data curation: A.P., C.R., and V.C. Resources: V.C. and C.R. Visualization: A.P. and N.J. Writing—original draft preparation: A.P. and C.R. Writing—review and editing: A.P., C.R., V.C., N.J., and T.L. Supervision: A.P. and T.L. Project administration: T.L. Funding acquisition: T.L. and C.R. A.P. and V.C. have contributed equally to this work.

## Funding

Open access publishing was facilitated by the Université de Genève, as part of the Wiley–Université de Genève agreement via the Consortium of Swiss Academic Libraries.

## Disclosure

All authors have read and approved the final version of the manuscript. Alexandre Perez had full access to all of the data in this study and takes complete responsibility for the integrity of the data and the accuracy of the data analysis.

## Ethics Statement

The presented study adhered to the Helsinki Declaration of Ethical Principles by the World Medical Association. This study did not require the approval of the Ethics Commission on Human Research of Geneva (CCER‐Geneva) according to the Federal Human Research Act (Art.3al.a) since the study involved one patient and does not involve experimental intervention or the use of identifiable patient data.

## Consent

Written informed consent was obtained from the patient for the publication of this article and accompanying images.

## Conflicts of Interest

The authors declare no conflicts of interest.

## Data Availability

No new data were created or analyzed in this study. Data sharing is not applicable to this article.
